# Modulation of Gonadotropins Activity by Antibodies

**DOI:** 10.3389/fendo.2019.00015

**Published:** 2019-02-18

**Authors:** Elodie Kara, Laurence Dupuy, Céline Bouillon, Sophie Casteret, Marie-Christine Maurel

**Affiliations:** ^1^Igyxos SA, Nouzilly, France; ^2^Service de Médecine et Biologie de la Reproduction, CHRU de Tours, Tours, France; ^3^Biologie Intégrative de l'Ovaire, INRA, UMR85, Physiologie de la Reproduction et des Comportements, Nouzilly, France; ^4^CNRS, UMR7247, Nouzilly, France; ^5^Université François Rabelais, Tours, France; ^6^IFCE, Nouzilly, France

**Keywords:** potentiating antibodies, inhibitory antibodies, follicle-stimulating hormone, luteinizing hormone, chorionic gonadotropin, signaling

## Abstract

Gonadotropins are essential for reproduction control in humans as well as in animals. They are widely used all over the world for ovarian stimulation in women, spermatogenesis stimulation in men, and ovulation induction and superovulation in animals. Despite the availability of many different preparations, all are made of the native hormones. Having different ligands with a wide activity range for a given receptor helps better understand its molecular and cellular signaling mechanisms as well as its physiological functions, and thus helps the development of more specific and adapted medicines. One way to control the gonadotropins' activity could be the use of modulating antibodies. Antibodies are powerful tools that were largely used to decipher gonadotropins' actions and they have shown their utility as therapeutics in several other indications such as cancer. In this review, we summarize the inhibitory and potentiating antibodies to gonadotropins, and their potential therapeutic applications.

## Introduction

Gonadotropins, namely follicle-stimulating hormone (FSH), luteinizing hormone (LH), and chorionic gonadotropin (CG) are heterodimeric glycoproteins, constituted by an alpha- and a beta- subunit. The alpha subunit is common to all glycoprotein hormones, including thyroid stimulating hormone (TSH) (1). FSH and LH/CG receptors are G-protein coupled receptors (GPCR), mainly expressed in granulosa cells in female ovaries and in Sertoli cells in male testis for FSH receptor (FSH-R) (2), and in granulosa and theca cells in female and Leydig cells in male for LH receptor (LH-R) (3).

Because of their role in reproduction, gonadotropins are routinely used in fertility treatments in men and women for assisted reproductive technologies (ART) (4, 5). In women, it consists on daily injections of FSH or a mixture of FSH/LH, for 8–12 days, to grow and mature follicles. The final maturation is then completed with an injection of human CG (hCG) 36 h after the last injection of FSH. In men, FSH and hCG injections 2–3 times a week for several months are used to treat hypogonadotropic hypogonadism and induce spermatogenesis (6–9). Currently, the preparations used are either endogenous FSH extracted from post-menopausal women urine (human menopausal gonadotropins, hMG), highly purified urinary FSH, or recombinant FSH. The first recombinant versions of FSH (follitropin alpha and beta, corifollitropin) and all their biosimilars were produced in Chinese hamster ovary (CHO) cells. The follitropin delta approved in Europe in 2015 (Rekovelle™) and follitropin epsilon still under development are produced in human cell lines: PER.C6 for follitropin delta (10) and GlycoExpress for follitropin epsilon (11). For LH and hCG, recombinant versions produced in CHO cells are also available. In animals, equine CG (eCG), formerly named pregnant-mare serum gonadotropin (PMSG), is widely used to induce ovulation in small ruminants (12). Porcine pituitary extracts are used for current superovulation treatments in cattle (13), whereas ovulation in swine herds is induced with a mixture of hCG and eCG (14).

Despite all the preparations that are on the market, the only ligands available for gonadotropin receptors as therapeutics are native hormones. New biased ligands or ligands with different potencies and efficacies on gonadotropin receptors can help better understand receptor signaling, decipher the implication of the different signaling pathways in physiological and pathophysiological mechanisms, and finally bring to the market new molecules to improve ART treatments. In 2015 in USA, 182,111 ART procedures were performed leading to 59,334 live-birth deliveries (15). Among other strategies like small molecule ligands, one way to modulate the gonadotropins' activity could be the use of antibodies, targeting either directly the receptor itself, or its ligand to modulate hormone's activity.

Antibodies are useful tools that help to better understand gonadotropins' structure by epitope mapping (16–18) and their function by neutralizing the effect of endogenous gonadotropins (19–22). They also allow their quantification by the development of radioimmunoassays (RIA) and enzyme-linked immunosorbent assays (ELISA) (23–27). Finally, antibodies permitted the development of specific purification methods for gonadotropins, making them safer to use as therapeutic agents (28–30).

The effect of antibodies, if any, is expected to be inhibitory on antigen activity by impairing its interaction with its receptor. Surprisingly, some of them were described as being able to increase the activity of their antigenic protein. Potentiating polyclonal antibodies directed against epidermal growth factor (EGF) and insulin were first described by Shechter et al. in 1979 (31, 32). A few years later, potentiating monoclonal antibodies (mAbs) were described for ovine (o) and human (h) growth hormones (GH) (33–35). When hypopituitary Snell dwarf mice were treated with hGH in complex with a mAb, the actions of hGH on growth and body composition were enhanced compared to animals treated with GH alone (34, 35). The same kind of antibodies were described for TSH: mAbs directed against TSH were able to enhance its biological activity *in vivo* in Snell dwarf mice (36), suggesting that glycoprotein hormones' activity may be modulated by mAbs. Anti-receptor antibodies with stimulating activities were also described for TSH receptor (37, 38).

In this review, we give a brief overview of antibodies modulating gonadotropins' activity, either positively or negatively.

## Structure and Function of Gonadotropins

Alpha- and beta-subunits of gonadotropins are non-covalently linked. The alpha-subunit is common to all glycoprotein hormones in a given species, and presents two major sites of N-glycosylation (1). The specificity of each hormone is conferred by the beta-subunit, that contains 2 N-glycosylation sites. hCG, eLH and eCG present a longer beta-subunit with an additional carboxy-terminal peptide (CTP) that is ~30 amino-acids long and contains multiple O-glycosylation sites.

eCG originates from uterine endometrial cups and is extracted from pregnant mare serum (39, 40). eLH and eCG beta-subunits are encoded by a single gene (41) but they differ in glycosylation. They both exhibit N-glycans on alpha- and beta-subunits, and O-glycans on the carboxy-terminal peptide (CTP) constituted of the last 29 amino-acids of the beta-subunit (beta 121–149) (42). With a carbohydrate content higher than 40% (43) and N-glycan chains terminated by sialic acids, eCG is the most heavily glycosylated glycoprotein hormone and has a longer *in vivo* half-life than other gonadotropins (~60 h) (44, 45). eCG binds to LH receptors in equine, but exerts FSH and LH actions in non-equine species by stimulating FSH and LH receptors respectively (46–51). Its dual FSH/LH activity and its longer half-life were the reasons why eCG was widely used since decades to induce ovulation in breeding animals, especially in goats and ewes for out-of-season breeding to allow artificial insemination.

hCG is mainly produced during pregnancy, by syncytiotrophoblast cells and a hyperglycosylated isoform is produced by cytotrophoblast cells. These two isoforms are implicated in implantation and early embryo development. Regular hCG for example promotes progesterone secretion by corpus lutea, angiogenesis of uterine vasculature, or growth, and differentiation of fetal organs, whereas hyperglycosylated hCG stimulates implantation by invasion of cytotrophoblast cells or stimulates growth of placenta (52). However, hCG can also be produced in non-pregnant women: it is produced at low levels by gonadotrope cells of the anterior pituitary, and seems to have an LH-like activity during menstrual cycle. Free beta-subunit of hCG is produced by multiple non-trophoblastic cancers. It is elevated in most cancers such as bladder, renal, prostate, gastrointestinal, lung, breast, neuroendocrine, gynecological, head and neck, and hematological cancers (53) and promotes their growth and malignancy by blocking apoptosis in cancer cells (52). hCG beta-subunit is thus used as a tumor biomarker usually associated with poor prognosis (53).

hCG, unlike eCG, has an LH like activity only and does not bind FSH-R (1, 54). CG and LH exert their effects *via* the same receptor, LH/CG receptor (LHCGR), that is coupled to Gs and Gq in granulosa cells and theca cells (55). However, the receptor is able to differentiate the binding and activity of these two hormones (56), and hLH and hCG differentially regulate signaling pathways (57, 58).

FSH plays an important role in reproduction. In females, it is implicated in follicular growth. FSH-R is expressed in granulosa cells and it is mainly coupled to Gs, which activates adenylyl cyclase and induces the secretion of cyclic adenosine monophosphate (cAMP), but it is also known to be coupled to Gq. In males, FSH regulates spermatogenesis. FSH-R is expressed in Sertoli cells and signals *via* Gs and Gi (59). In HEK 293 cells expressing FSH-R, FSH stimulates Gs/cAMP/PKA signaling pathway as in granulosa or Sertoli cells, but also signals *via* a beta-arrestin-dependent pathway, leading in both case to extracellular signal-regulated kinases (ERK) 1/2 phosphorylation but with different kinetics (60).

FSH-R is also expressed in other tissues than reproductive organs, such as osteoclasts (61) or adipose tissue (62), suggesting that FSH may play other physiological roles. In adipose tissue, FSH-R is coupled to Gi. An increase in Ca2+ influx induces the phosphorylation of cAMP-response-element-binding protein, which in turn activates an array of genes involved in fatty acids and triglycerides biosynthesis (63). FSH is thus implicated in lipid biosynthesis and its storage in adipocytes, which may contribute to age-related obesity and diseases due to high FSH levels in aging populations (62, 63). The first paper mentioning the role of FSH in bone mass regulation was published in 2006 (61). The authors proposed a mechanism where FSH was able to increase osteoclasts formation and function via a Gi2a-coupled FSH-R expressed in these cells and their precursors (64), suggesting that high circulating FSH levels were responsible for post-menopausal osteoporosis.

## Antibodies Modulating the Activity of Chorionic Gonadotropins

### Antibodies Modulating the Activity of eCG

Most of the antibodies against eCG were developed for structural analysis purposes. Maurel et al. identified an antibody able to inhibit eCG binding to LH and FSH receptors (65). Chopineau et al. analyzed the affinity and the specificity of 14 mAbs directed against eCG (66). The aim of this study was to analyze the epitopic sites of eCG and permitted to draw a topographic map of antigenic and functional sites of this hormone. The affinity of antibodies for eCG was ranging between 10^−7^ and 10^−11^ M. Ten of them were alpha-subunit specific because they recognized both native eCG and free alpha-subunit, but not free beta-subunit. One antibody exhibited a higher affinity for alpha-subunit than for the native eCG, and 13 mAbs exhibited a better affinity for the dimer than for the free subunits. The effect of these mAbs was then tested on FSH and LH bioactivities of eCG in *in vitro* bioassays. One beta-subunit specific, one alpha-subunit specific and one native alpha/beta dimer specific antibodies did not show any effect on FSH and LH bioactivities. Nine alpha-subunit specific antibodies either weakly or strongly inhibited both bioactivities. Finally, two mAbs were potentiating FSH bioactivity of eCG: one was beta-subunit specific and the other was native dimeric eCG specific. They did not inhibit eCG binding to LH-R. The degree of inhibition of inhibitory antibodies was correlated with their affinity for eCG, but it wasn't for the two antibodies potentiating FSH bioactivity of eCG. These data suggest that the inhibitory or potentiating effects of mAbs on eCG bioactivities neither depend on their specificity nor their affinity. Moreover, the two antibodies potentiating the FSH bioactivity of eCG were either not affecting or inhibiting weakly the LH bioactivity of eCG, demonstrating that the effect on both bioactivities can be opposite (inhibitory or potentiating), with different degrees of activity (none or weak) on hormone bioactivities, highlighting the potential multiple mode of action of antibodies.

The high carbohydrate content makes also eCG highly immunogenic. Repeated injections of eCG for ovulation induction decrease the fertility of goats from 60 to 40% (12, 67). Roy et al. detected an immune response in animals treated with eCG for ovulation induction (68, 69), and demonstrated that the secreted antibodies from a previous treatment were inhibiting the action of eCG injected for the following treatment. The goats with high antibody levels at the time of eCG administration did have a much lower kidding rate (41%) than the other females (66%). This immune response was thus altering the fertility of these animals by delaying both the onset of estrus and the preovulatory LH surge. However, a deeper analysis revealed that some of the goats secreting high levels of anti-eCG antibodies did have a fertility beyond expected, ovulating and getting pregnant after each treatment, even after four treatments. The antibodies from the plasma of hypo-fertile or hyper-fertile goats secreting high levels of antibodies were purified and the IgG fractions were analyzed for their effect on FSH bioactivity in Y1 cell line derived from a mouse adrenal cortex tumor stably expressing human FSH-R, and for their effect on LH bioactivity in rat Leydig cells. The plasmas and the corresponding IgG fractions from different eCG treated goats exhibited either inhibitory, enhancing or no effects on FSH activity of eCG by modulating progesterone secretion by Y1 cells, and on LH activity of eCG by modulating testosterone secretion in Leydig cells (70). As expected, antibodies were mainly recognizing carbohydrate chains of eCG. Twenty-one either inhibitory, potentiating or neutral antibodies for LH and/or FSH bioactivities of eCG were analyzed. None of the antibodies cross-reacted with totally deglycosylated eCG or alpha-subunit of eCG. Interestingly, the inhibitory or stimulatory effects of these antibodies were not correlated with the affinity of the tested antibody for eCG (70). All together, these data demonstrated that gonadotropins' *in vivo* bioactivity and animals' fertility can be modulated with antibodies, especially since these antibodies are naturally occurring.

Later, Wehbi et al. (71) analyzed the effect of eCG in complex with three of these antibodies on FSH receptor signaling pathways. The eCG/antibody complexes effect was tested on HEK 293 cells expressing mouse FSH receptor and on granulosa cells punctured from slaughterhouse cows for their effect on cAMP production. The tested antibodies were differently modulating cAMP production: two of them were potentiating and one was slightly inhibiting eCG effect. In contrast, all three antibodies were enhancing ERK1/2 phosphorylation in HEK 293 cells expressing mouse FSH-R. Deeper analysis revealed that the antibodies were potentiating eCG signaling preferentially *via* beta-arrestin pathway, *via* cAMP/PKA pathway or *via* both. An antibody complexed to eCG was thus able to change the full agonist effect of eCG into a biased agonist effect, modulating differentially the balance between the signaling pathways induced by this hormone. This paper was also the demonstration that these antibodies were achieving the same *in vivo* effect in goat (i.e., high prolificity) *via* different signaling pathways. That was the first report of biased agonism at FSH-R and the authors suggested that such antibodies could help optimize glycoprotein hormones' bioactivities and thus the development of new therapies.

At the very beginning of infertility treatments, eCG was also used to treat women. The first successful treatment with eCG was described in 1945. Although its use lasted more than 30 years, scientists realized very early that women treated with eCG extracts, like animals, did produce “antigonadotrophic substances” which neutralize hormone's effect over time and after repeated injections. The immune response induced by eCG and the arrival of less immunogenic pituitary gonadotropin extracts led to the market withdrawal of eCG [reviewed in (4)].

### Antibodies Modulating the Activity of Human Chorionic Gonadotropin (hCG)

As for other gonadotropins, anti-hCG antibodies were essentially developed for epitope mapping and variant specific mAbs permitted the development of immunoassays, leading *in fine* to pregnancy tests (72–74).

Naturally occurring endogenous antibodies were also reported: patients treated with exogenous gonadotropins can develop anti-hCG antibodies that impair fertility. They were detected in young men with hypogonadotropic hypogonadism treated with hCG (75, 76). These antibodies, detected in a 15 year-old patient following a secondary resistance during a third treatment to hCG, were low affinity but high binding capacity antibodies (76). A few years later, seven additional young men with hypogonadotropic hypogonadism, aged between 11 and 18 years, and resistant to classical hCG regimen were tested for the presence of anti-hCG antibodies. Four of them showed antibodies, but the neutralizing effect of hCG could be counter passed by increasing the doses of hCG used (75). The same kind of antibodies were described in women (77, 78). Immune response against hCG impairs fertility of women and induces pregnancy loss within the first trimester of pregnancy. To thwart this negative effect, Muller and collaborators described a treatment that was successful in three women positive for hCG antibodies. This treatment combined membrane plasmapheresis, prednisolone, and intravenous immunoglobulin therapy (78).

Anti-hCG auto-antibodies were also detected in sera of men and women that never received any injection of exogenous hormone (79). These antibodies were low affinity and did not interfere with hormone activity. However, few years later, antibodies with high affinity and the capacity to neutralize hCG and LH activities were detected in a patient with a history of spontaneous abortion, that was never exposed to exogenous hormone therapy (80).

## Antibodies Modulating the Activity of FSH or LH

### FSH and/or LH Neutralization With Antibodies

Several inhibiting antibodies were described for FSH and most of them were used to better understand its physiological functions *in vivo*. Antibodies permit to block reversibly the action of one or several hormones, at a precise time of the lifespan of the studied model, rather than suppressing a whole organ like in hypophysectomy or a gene like in transgenic animals. For example, for female studies, in 1969, Goldman and Mahesh used an anti-sera obtained by rabbit immunization with ovine LH, that neutralized FSH as well as LH, to study the role of these hormones in ovulation (81). In 1970, the same group published data on the effect of the same anti-sera on neonatal rats (82). In 1971, Eshkol and Lunenfeld used the strategy of neutralization with antibodies to demonstrate the crucial role of FSH and LH in ovarian development during the first 2 weeks of life in rodents. FSH was found to be responsible for the stimulation of granulosa cell proliferation, organization and structure. FSH plus LH initiated secretory activity of granulosa cells, increased intrafollicular spaces, antrum formation, enrichment and maintenance of the theca layer, and development of the vascular system (19). At the same time, it was shown that LH anti-sera could block ovulation in rat, but not FSH anti-sera (83–86). Schwartz et al. suggested that FSH neutralization during estrus cycle could have a deleterious effect on follicles destined to grow and ovulate in following cycles (86). Several other studies have confirmed the role of LH as the indispensable trigger of ovulation, whereas FSH was required for the recruitment of antral follicles at the start of a new cycle in rat and hamster (87–91). The neutralization of FSH or LH with antibodies also permitted to study the role of these hormones in the synthesis of estrogen (92), on ornithine decarboxylase activity (93) and on gonadotropin surge-inhibiting factor/attenuating factor bioactivity (20) in rat and/or hamster. In monkey, the antibody neutralization of FSH highlighted the importance of FSH during follicular growth and showed that the mature follicle becomes less sensitive to FSH about 48 h before ovulation (94).

For male studies, Wickings and Nieschlag actively immunized *Macaca mulatta* against ovine FSH and observed a spermatogenesis suppression over a period of 2 years, confirming the importance of FSH for spermatogenesis (21). The importance of FSH in spermatogenesis was further confirmed by active immunization of *Macaca radiata* (95). In rat, even if the first studies obtained with immunoneutralization of FSH were controversial on the role of FSH in spermatogenesis (96, 97), later works with either passive (98) or active (22) immunization against FSH confirmed its crucial role in the maintenance of spermatogenesis. For active immunization, peptides from region 19–36 of rat FSH beta-subunit were used (22). Altogether, these results suggest that immunoneutralization of FSH could be used as a contraception in men by suppressing spermatogenesis. However, Nieschlag recommended to abandon the approach of immunization as a contraception because a complete suppression of spermatogenesis was not achieved even after 4.5 years of immunization (99). Nevertheless, Moudgal and collaborators carried out a pilot study in 1997 where five male volunteers were immunized with ovine FSH isolated from sheep pituitaries (100). The subjects only responded to the first two immunizations (day 1 and 20), and did not respond to the boosters given at day 40 and 70. Ovine FSH vaccination generated antibodies against human FSH, but only 25–45% of the antibodies generated against ovine FSH were able to bind human FSH and the sperm count reduction was around 30–64%, which is not enough to consider this method as a contraception.

Anti-FSH antibodies were also detected in women. Two types of antibodies have been identified: naturally occurring anti-FSH antibodies (101, 102) and anti-FSH antibodies resulting from exogenous gonadotropins (103–106). First, Haller et al. found naturally occurring anti-FSH antibodies in patients with endometriosis or polycystic ovary syndrome (PCOS) and none of these patients had undergone ovarian stimulation for IVF. They also detected anti-FSH antibodies in healthy non-pregnant women but at lower rates than for patients with endometriosis or PCOS (101). Likewise, Shatavi et al. found spontaneous anti-gonadotropin antibodies in 27% of patients with unexplained infertility and never treated with gonadotropins, but only in 8% of women in the general population (102). To explain the presence of such spontaneous antibodies, it was supposed that an alteration of the immune system might be necessary and that the antigen responsible for their production could be either the circulating FSH from the female organism or the FSH in seminal fluid that may upregulate the anti-FSH immune response in females (101). In *in vitro* fertilization (IVF) patients, Haller et al. demonstrated that anti-FSH antibodies increase in infertile women with common autoantibodies (against nuclear antigens, smooth muscle, gastric parietal cells, b2-glycoprotein I, cardiolipin, and thyroid peroxidase) and with a history of IVF stimulation (103, 104). Shatavi et al. also found that anti-FSH antibodies were more recurrent in infertile patients with history of gonadotropin treatment than in infertile patients never treated with exogenous FSH or in women in the general population (102). Anti-FSH antibodies could also be associated with anti-ovarian antibodies (AOA) in patients with history of gonadotropin treatment (102, 107). The association of anti-FSH antibodies and AOA was also detected in infertile patients never treated by FSH and in women in the general population but at a lower frequency (102). Other studies have investigated the consequences of the presence of anti-FSH antibodies on the results of controlled ovarian stimulation (COS). Some studies found that anti-FSH antibodies were associated with poor ovarian response to IVF stimulation (104, 105). Thus, anti-FSH antibodies might have an inhibitory effect on FSH by preventing the binding of the hormone to its receptor or by trapping FSH in immune complexes (101, 107). On the contrary, Reznik et al. identified a higher proportion of anti-FSH antibodies in patients with a good response to COS compared to patients with a poor response (106). Antibodies produced in patients with a good response might have either no effect or a potentiating effect on the action of FSH. However, in humans, no *in vitro* study of the inhibitory or potentiating action of anti-FSH antibodies on FSH receptor signaling has been published yet. In human FSH, one of the major epitopes seems to be the 78–93 amino acid sequence of the β-chain (101, 107). This region contains a loop called cysteine noose which plays a role in the specificity of FSH receptor binding (101). Therefore, it was supposed that the binding of the hormone to its receptor could be modulated by antibodies directed against this region (101). To explain why some infertile patients develop anti-gonadotropin antibodies, some studies focused on the Major Histocompatibility Complex (MHC) Class II (103). The role of the MHC Class II is to present exogenous proteins to immune cells, which leads to a humoral immune response. In IVF patients, only anti-FSH IgA were associated with HLA-DQB1^*^03 (103). However, the development of anti-FSH antibody response to exogenous FSH treatment remains controversial. Indeed, a recent study conducted in healthy oocyte donors and infertile women has concluded that repeated gonadotropin treatments for IVF do not induce an immune response to FSH (108).

### Antibodies Potentiating the Activity of FSH

The first anti-FSH mAbs described were directed against human FSH (16). Their binding specificities were well-characterized, but their effect on FSH activity was not investigated. The second anti-FSH mAb described in the literature was directed against bovine FSH (29) and was beta-subunit specific. It did not cross react with ovine or porcine FSH, indicating that it recognizes an area of bovine FSH not homologous to ovine or porcine hormones (29). Glencross et al. tested this antibody later on for its effect on bovine FSH bioactivity in hypopituitary Snell dwarf mice (109). The mAb injected in complex with FSH increased uterine weight whereas FSH alone or the mAb alone at the same concentrations did not have any effect, showing for the first time a potentiating effect with an anti-FSH mAb.

Holder's group described anti-sera directed against peptides derived from the beta-subunit of bovine FSH (110). When injected to hypopituitary Snell dwarf mice concomitantly with ovine FSH, these anti-sera produced by sheep immunized with peptides corresponding to 31–45 and 38–49 amino-acid regions of bovine FSH beta-subunit were able to enhance FSH activity, as measured by an increase in uterine and ovarian weight, and an increase in the percentage of keratinized cells in vaginal smears. The authors hypothesized that the administration of anti-peptide anti-sera precomplexed with FSH or active immunization of breeding animals with these peptides should result in a superovulatory response, and that the potentiating anti-sera strategy, in the case of FSH, could be used for several clinical situations, such as treatment of ovarian disorders related to low FSH secretion, induction of estrus and treatment leading to increase spermatogenesis.

## Anti-Gonadotropin Antibodies as Therapeutic Agents

### Therapeutic Antibodies in General

MAbs, initially developed for scientific purposes, now take part of the human therapeutic arsenal. Over the last three decades, they have grown to become more than 55% of the overall biotherapeutic market of the drug industry sales (111).

The regulatory story of therapeutic mAbs started in 1986 with the first FDA-approved therapeutic mAb, the murine mAb Orthoclone OKT3® (Muromonab CD3) indicated for the prevention of kidney transplant rejection. Unfortunately, the development of murine mAbs has been hindered because of the risk of immunogenicity in humans due to their murine elements. Replacing the constant region of murine mAb by human sequences resulted in the generation of the chimeric antibodies (~30% murine content). The first-approved one, Rituxan® (Rituximab) in 1997, was used for the treatment of low grade B cell lymphoma. To overcome immunogenicity risk even further, new technologies for the generation of predominately or entirely human origin mAbs were developed. The humanized mAbs (5–10% murine content) are tailored by replacing all sequences by human except antigen binding complementary determining regions, which were derived from the mouse. The humanization technology developed by Sir Winter lead to the first humanized therapeutic antibody CAMPATH-1H® (Alemtuzumab, approved in 2001). Sir Winter was awarded the 2018 Nobel Prize in Chemistry along with George Smith for this technology and the fully humanization using phage display. Fully human mAbs (last generation) were developed by replacing the whole of the rodent sequences by human sequences. Humira® (Adalimumab) is the first fully human antibody approved in 2004, for the treatment of rheumatoid arthritis. Thanks to these new technologies, the rate of approval and mAbs available on the market for the treatment of various diseases has increased dramatically. In 2017, the FDA and the European Medicines Agency broke the record and approved 10 new therapeutic antibodies (112). Currently, more than 70 mAb products are available on the market, most of them being humanized (32%) or fully human (54%) (113).

To date, approved mAbs are from different isotypes, but the preferred mAbs in clinical use are of the IgG1 isotype (80%) (114). Additionally, there are five monovalent antibody-fragments on the market, four antigen-binding fragments (Fab) and 1 single chain variable fragments (scFv) (114). More sophisticated forms have been engineered, such as Fc-modification, IgG2/IgG4 hybrid Fc, glyco-engineered mAbs, bispecifics, or antibody-drug conjugates. These types of sophisticated mAbs reach more and more clinical trial studies (114, 115). According to Zhou and Mark (116), common mechanism of action proposed for mAb drugs include: (i) disruption of ligand–receptor interaction; (ii) target cell elimination via antibody-dependent cellular cytotoxicity (ADCC), complement-dependent cytotoxicity (CDC), and antibody-dependent cellular phagocytosis (ADCP); (iii) engagement of cytotoxic T cell by bispecific Abs; (iv) receptor downregulation by enhanced internalization and degradation; and (v) targeted drug delivery.

The indication of treatment for ~80% of the therapeutic mAb drugs could be classified into oncology and immune diseases. The last ~20% are used for the treatment of infection and cardiovascular diseases, orthopedic, eye and rare diseases (113). Despite the high treatment cost, the success of therapeutic mAb has recently reached the veterinary health with the launch in the European Union, in 2017, of Cytopoint® (Lokivetmab), a treatment for atopic dermatitis in dogs. Notwithstanding all the therapeutic mAbs developed to date, so far, none of them have succeeded in the field of fertility, and none of them are potentiating antibodies.

### Therapeutics Involving Anti-gonadotropin Antibodies

#### Neutra-PMSG®, an Antibody Against eCG/PMSG

The unique antibody commercialized until now in the field of animal fertility is Neutra-PMSG®. This anti-eCG mAb is alpha-subunit specific and inhibits both LH bioactivity on small bovine luteal cells and FSH bioactivity on granulosa cells of bovine follicles (66). It was developed and marketed for cattle to limit the adverse effects of PMSG to improve the embryo production after superovulation treatment (117, 118). Due to a long half-life, PMSG had the disadvantage to cause a prolonged stimulation of follicular growth after preovulatory LH peak, inducing a poor response to superovulation treatment for cattle (117, 119, 120), sheep (121), and goat (122). Neutra-PMSG® injected 1–2 days after PMSG in superovulation treatment neutralized the adverse effects of PMSG by reducing its half-life in systemic circulation, improving embryo production. This mAb did not recognize endogenous gonadotropins in treated animals. Therefore, Neutra-PMSG® was highly specific for PMSG (117). Currently, most cows are superovulated using pituitary extracts containing FSH and LH (13), mainly because the pharmaceutical company (Intervet, The Netherlands) that developed the Neutra-PMSG® mAb stopped production in the 1990s.

#### Active Immunization Against hCG

A vaccination against hCG was also considered in the 1970s as a contraception method to avoid pregnancy in women (123, 124). The aim is to induce the secretion of antibodies that will bind hCG and block its activity, thus impeding pregnancy. Because an immunization with the whole dimeric hCG (alpha+beta-subunits) was raising antibodies not only against hCG but also against human LH (125), a special preparation of beta-hCG was made by processing it against heterologous anti-LH immunosorbents (124) and conjugating it to purified tetanus toxoid as an immunogenic carrier (123). This processed molecule was able to induce an immune response with antibodies specific to hCG. The antibodies produced were able to abrogate the binding of hCG to its receptor and its biological effects in *in vivo* bioassays. Moreover, the antibody titer declines over time, indicating that the vaccination is reversible (126). This vaccination system went through phase 1 clinical trials in several countries (India, Finland, Sweden, Chile and Brazil) under the International Committee on Contraception Research of Population Council. A slightly different preparation consisting of a dimer of hCG beta-subunit non-covalently associated with ovine LH alpha-subunit conjugated to tetanus and diphtheria toxoids (127) underwent phase 1 and phase 2 clinical trials in several centers in India. Eighty percent of treated patients generated sufficient antibody titer (>50 ng/ml) to be protected against pregnancy, and only one pregnancy was recorded over 1,124 cycles in fertile and sexually active women with an antibody titer higher than 50 ng/ml. After 12 years of inactivity on this project, Talwar and his collaborators are now working on a genetically engineered recombinant vaccine that is expected to go through clinical development in the next few years (128).

A similar approach was also tested for colorectal and pancreatic cancer treatments. In 2000, AVI BioPharma collaborated with SuperGen for the clinical development and marketing of Avicine, a synthetic vaccine constituted of the C-terminal peptide of hCG (CTP-37) conjugated to diphtheria toxoid. The vaccine went through several clinical trials until phase 3 pivotal study. In phase 2 efficacy study for colorectal cancer, 73% of treated patients developed an immune response against hCG and this response was associated with an improved median survival time (129, 130). As far as we know, the product has not reached the market yet. Recently, another group proposed another vaccine, where one residue in the amino-acid sequence of hCG beta-subunit is substituted (hCGβR68E) to eliminate cross reactivity with LH and conjugated to heat shock protein (Hsp70) as carrier to increase its immunogenicity (131). This vaccine has not been tested in humans yet.

### Other Potential Therapeutic Antibodies

#### Antibodies Targeting FSH

To consolidate the assumption that high circulating FSH levels were responsible for post-menopausal osteoporosis, Zhu et al. showed that blocking FSH action attenuates bone loss in ovariectomized mice *via* two mechanisms: by inhibiting bone resorption and by stimulating bone formation (132). To do so, they used a mouse polyclonal antibody targeting a 13-amino acid sequence (LVYKDPARPNTQK) of mouse FSH beta-subunit that is a receptor-binding domain.

Liu et al. hypothesized that a pharmacological blockade of FSH action could also reduce body fat mass. In fact, they have shown that the same polyclonal antibody targeting the receptor-binding domain of FSH beta-subunit, injected daily for 8 weeks in mice, prevented the gain of body fat induced by the diet in male and female mice (133). This decrease of body fat was associated with an increase of fat thermogenesis (133, 134). For the purpose of potential therapeutic application in human, this team has developed a mAb targeting the same epitope in human FSH beta-subunit (LVYKDPARPKIQK) that had the same effects on body fat and thermogenesis on the mouse as the mouse polyclonal antibody directed against a sequence that is 2 amino-acids different (LVYKDPARPNTQK) (135, 136). The modulation of FSH activity by anti-FSH antibodies may be considered as therapeutic means to reduce the risk of obesity in elderly people with high levels of FSH (133). It was thus proposed that the same antibody could both inhibit bone loss and body fat gain (133, 136). However, while some of the other studies published on the subject supported the role of FSH in bone mass regulation (137–141), some others were contradictory (142, 143). Moreover, clinical studies on human subjects reported that FSH suppression with GnRH agonist had no effect on bone resorption in women (144). In men, this suppression either increased bone loss (145), or had no effect when a testosterone supplementation was given (146). Furthermore, body fat mass is also increased in men treated with GnRH analog (147). Altogether, these data suggest that further investigations are needed to better understand the mechanisms underlying the role of FSH in bone mass and body fat regulation before a therapeutic approach can be envisaged (148). A therapeutic antibody to prevent osteoporosis is on the market since 2010. Receptor activator of nuclear factor-kB (RANK) ligand (RANKL) is a cytokine necessary for the development and the activity of osteoclasts. A fully human antibody, denosumab (Prolia®, Amgen) prevents RANKL binding to its receptor RANK. Denosumab, when given subcutaneously twice yearly for 36 months, reduces the risk of vertebral, non-vertebral, and hip fractures in women with osteoporosis (149), demonstrating that the strategy of therapeutic antibody can be used in this indication.

#### Antibodies Targeting hCG

Antibodies able to inhibit hCG activity were first described in 1980 (150). They were specific of the beta-subunit of hCG and did not cross react at all with other gonadotropins. Recently, one of these antibodies (mAb PIPP) was expressed recombinantly in tobacco leaves in different formats (scFv, diabody and entire antibody) and tested, after their extraction and purification, for their efficacy to neutralize hCG. The three formats of the same antibody were able to inhibit *in vitro* testosterone production induced by hCG in Leydig cells. *In vivo*, the entire mAb was able to block uterine weight gain in mouse model (151). These antibodies were envisaged as a contraception method by passive immunization in women, and were considered as a better method than active immunization where the response may be variable between patients, and a sufficient titer determined as the protective level of antibody was observed in 80% of the patients only. These anti-hCG antibodies have not entered a clinical development so far.

The same antibody was used for the development of an immunotoxin targeting hCG-expressing cancer cells (152). The VH and VL domains of the full antibody were linked together to form the scFv fragment (scFv PIPP). This scFv's gene was then fused with a gene expressing Pseudomonas exotoxin (PE38) and expressed in Escherichia coli as a recombinant protein (scPiPP-PE38). Once purified and tested on cancer cells, the immunotoxin showed 90% killing of hCG beta expressing histiocytic lymphoma, T-lymphoblastic leukemia, and lung carcinoma cells *in vitro*. However, further studies are needed to evaluate the potential of scPiPP-PE38 as a therapeutic agent for management of cancer cells expressing hCG or its subunits.

## Conclusion

Many different antibodies against gonadotropins were developed and have proven to be very useful tools for many applications. They can also be naturally secreted due to a humoral immune response to endogenous or exogenous gonadotropins. With the same structure, immunoglobulins can have inhibitory or potentiating effects depending on their paratope defined by CDRs and their epitope (binding site) on the antigen. In the case of eCG, its naturally occurring potentiating antibodies have demonstrated that a differential activation of signaling pathways of FSH-R could lead to the same *in vivo* effect, i.e., high prolificity in goats (71). The development of antibodies with a range of modulating effects on the potency and the efficacy of FSH on its signaling pathways could help deciphering the importance of each pathway for FSH roles in reproduction, bone mass and body fat regulation. Moreover, these antibodies can represent potential therapeutics, targeting one pathophysiological or physiological condition in particular. Several applications for anti-gonadotropin antibodies have already been proposed and are under exploration, like osteoporosis, obesity, contraception, or cancer. All of these indications require inhibition of gonadotropins' action. On the other hand, in small ruminants, the naturally occurring anti-eCG potentiating antibodies induced a better fertility and prolificity demonstrating that it is possible to improve fertility by potentiating gonadotropins' activity during several estrus cycles, without any side effects. All these studies demonstrated that it is possible to target each gonadotropin very specifically despite their similarities.

To conclude, the development of antibodies modulating gonadotropins' activity could not only provide new tools to better understand their roles in different physiological processes, but could also bring to the market innovative drugs. Taking into account the state of the art and the clinical development time, there is a long way to go until a therapeutic antibody targeting a gonadotropin can reach the market.

## Author Contributions

All authors listed have made a substantial, direct and intellectual contribution to the work, and approved it for publication.

### Conflict of Interest Statement

EK, LD, SC, and M-CM were employed by Igyxos SA. The remaining author declares that the research was conducted in the absence of any commercial or financial relationships that could be construed as a potential conflict of interest.
